# Exploring NUP62’s role in cancer progression, tumor immunity, and treatment response: insights from multi-omics analysis

**DOI:** 10.3389/fimmu.2025.1559396

**Published:** 2025-03-03

**Authors:** Lihong Chen, Youfu He, Menghui Duan, Tianwen Yang, Yin Chen, Bo Wang, Dejun Cui, Chen Li

**Affiliations:** ^1^ Department of Gastroenterology, Guizhou Provincial People’s Hospital, Guiyang, Guizhou, China; ^2^ Department of Cardiology, Guizhou Provincial People’s Hospital, Guiyang, Guizhou, China; ^3^ Department of Critical Care Medicine, The First Hospital of China Medical University, Shenyang, Liaoning, China; ^4^ Department of Orthopaedics, Guizhou Second People’s Hospital, Guiyang, Guizhou, China; ^5^ Department of General Surgery, Guizhou Provincial People’s Hospital, Guiyang, Guizhou, China; ^6^ Department of Pharmacy, the First Affiliated Hospital of Guangxi Medical University, Nanning, Guangxi, China

**Keywords:** Nup62, immune, pan cancer, gastric cancer, immune cell infiltration

## Abstract

**Background:**

NUP62, a key component of the nuclear pore complex, is closely associated with cellular functions and cancer progression. However, its expression patterns, prognostic value, and relationship with tumour immunity and drug sensitivity across multiple cancers have not been systematically studied. This study used multi-omics analyses combined with experimental validation in gastric cancer to investigate the expression, functional characteristics, and clinical relevance of NUP62 in cancer.

**Methods:**

Data from TCGA, GTEx, and CPTAC databases were used to analyse the expression, mutation characteristics, and clinical associations of NUP62. Tools such as SangerBox, TIMER 2.0, and GSEA were employed to evaluate the relationship between NUP62 and the tumour immune microenvironment, as well as its involvement in signalling pathways. Immunohistochemistry and RT-PCR were used to validate the expression of NUP62 in gastric cancer tissues. PRISM and CTRP databases were utilised to assess the correlation between NUP62 expression and drug sensitivity.

**Results:**

NUP62 was significantly upregulated in multiple cancers and was associated with poor prognosis in cancers such as clear cell renal carcinoma (KIRC), lower-grade glioma (LGG), and adrenocortical carcinoma (ACC), while playing a protective role in others, such as bladder cancer (BLCA) and stomach cancer (STAD). Functional analyses showed that NUP62 is involved in cell cycle regulation, DNA damage repair, and tumour immunity. High NUP62 expression was significantly correlated with increased infiltration of immune cells, such as macrophages and T cells, and a higher response rate to immunotherapy. Drug sensitivity analysis identified NUP62 as a marker of sensitivity to various chemotherapeutic agents. Validation experiments demonstrated that NUP62 mRNA and protein levels were significantly higher in gastric cancer tissues than in adjacent normal tissues.

**Conclusions:**

NUP62 plays a critical role in multiple cancers and shows potential as a biomarker for cancer diagnosis, prognosis, and therapeutic response prediction. Its role in tumour immunity and signalling pathways highlights its potential as a target for immunotherapy and precision medicine.

## 1 Background

Cancer represents a major public health issue worldwide and is the second leading cause of death in the United States. In 2022, there will be nearly 20 million new cases of cancer, while 9.7 million people will die of cancer. About one in five men or women will develop cancer in their lifetime, while about one in nine men and one in 12 women will die of cancer (1). The rate of decline in cancer mortality has risen from about 1 percent per year in the 1990s, to 1.5 percent per year in the 2000s, to 2 percent per year between 2015 and 2020 (2).This trend reflects the increasing depth of human research into cancer, which is closely related to advancements in early cancer diagnosis and targeted therapy (3–5). Pan-cancer analysis, which integrates multiple databases, can aid in the identification of cancer biomarkers and therapeutic targets (6, 7).

Metabolic reprogramming is a hallmark of tumors, whereby tumors undergo reprogramming of nutrient acquisition and metabolic pathways to meet the bioenergetic, biosynthetic, and redox demands of malignant cells (8, 9). There exists a close interaction between metabolism and signaling pathways in cancer cells, and several signaling pathways associated with cell proliferation also regulate metabolic pathways that integrate nutrients into biomacromolecules (10). Consequently, certain cancer-related mutations enable cancer cells to acquire and metabolize nutrients in a manner conducive to proliferation, rather than efficiently producing ATP (11, 12). To fulfill the demands of proliferation, cancer cells utilize processes such as glycolysis, glutaminolysis, and fatty acid oxidation to meet their energy requirements and metabolic synthesis processes (13, 14). Studies have indicated that oxidative phosphorylation represents an emerging target in cancer therapy (15). Furthermore, immune cells within the tumor microenvironment (TME) exhibit metabolic shifts similar to the glycolytic metabolic profile, leading to competition for nutrients between cancer cells and tumor-infiltrating cells (16). On the other hand, metabolic disturbances characterized by hypoxia and elevated metabolite levels (especially lactate) in the TME can result in immunosuppression (17, 18). Simultaneously, metabolic dysregulation and imbalances in immune cells within the TME can drive immune evasion and compromise treatment outcomes (19).

The primary function of the nuclear pore complex (NPC) is to mediate transport between the nucleus and cytoplasm. Studies have shown that nucleoporins exert effects independently of the NPC in both nuclear and cytoplasmic compartments, directly regulating gene expression and participating in the regulation of development and the cell cycle (20). Nucleoporin 62 (NUP62), a structural component of the NPC, may mediate the localization of glucocorticoid receptors to the nucleus after binding to steroids (21). Additionally, NUP62 can interact with OSBP-related protein 8 (ORP8), which has the ability to regulate hepatic lipogenesis and plasma lipid levels (22). Furthermore, numerous studies have demonstrated that NUP62 mediates selective nucleocytoplasmic transport and is associated with various cancers through chromosomal translocations that generate fusion proteins, alterations in protein expression levels, and single-point mutations (23–27). Specifically, high expression of NUP62 contributes to preventing epidermal differentiation in squamous cell carcinomas originating from stratified epithelia (27, 28).

## 2 Materials and methods

### 2.1 Data collection and processing

We obtained expression levels and related clinical characteristics of NUP62 from the Cancer Genome Atlas (TCGA, http://cancergenome.nih.gov/) and the Genotype-Tissue Expression (GTEx, https://gtexportal.org/) databases through the Xena platform at the University of California, San Diego. The mutation frequency of NUP62 in the TCGA cohort was calculated using the cBioPortal database (https://www.cbioportal.org/). Pan-cancer analysis of TCGA samples was conducted using the online bioinformatics tool SangerBox 3.0 (http://sangerbox.com/) (29). Various immune infiltration algorithms in the TIMER 2.0 database (http://timer.cistrome.org) were utilized to characterize the correlation between NUP62 expression and the tumor immune microenvironment.

Expression profiles of human normal tissues and cancer cell lines were obtained from the Human Protein Atlas (HPA, https://www.proteinatlas.org/). Relevant chemotherapy data were retrieved from the Genomics of Drug Sensitivity in Cancer (GDSC, https://www.cancerrxgene.org/), Cancer Therapeutics Response Portal (CTRP, http://portals.broadinstitute.org/ctrp/), and PRISM databases to illustrate the relationship between NUP62 expression and drug sensitivity. Cancer immune cycle data originated from the Tracking Immune Phenotypes in Cancer (TIP, https://biocc.hrbmu.edu.cn/TIP/), while Immune Phenotype Scores (IPS) data were obtained from the TCIA website (https://tcia.at/home).

### 2.2 Expression and variation analysis

To investigate whether there were differences in NUP62 expression between tumor and normal tissues, we first compared the expression of NUP62 mRNA between tumor and normal tissues using the “wilcox” test. Subsequently, a paired “wilcox” test was conducted on matched samples to validate protein expression at the external gene transcriptome level in the GEO and CPTAC databases. Additionally, we used the “gganatogram” R package to visualize the expression of NUP62 in different human organs. The cBioPortal website (http://www.cbioportal.org) served as a powerful tool to search for NUP62 mutation frequency, types, copy number alteration (CNA) data, and gene alteration traits. Furthermore, we employed the “pROC” R package to calculate the Area Under the Curve (AUC) value, illustrating the importance of NUP62 in pan-cancer diagnosis.

### 2.3 Survival and clinical outcome analysis

Survival data were sourced from the TCGA database. We analyzed the relationship between NUP62 expression and these prognostic indicators, including Overall Survival (OS), Disease-Specific Survival (DSS), Progression-Free Interval (PFI), and Disease-Free Interval (DFI), using the “survival” and “survminer” R packages. Kaplan-Meier (KM) analysis and univariate COX analysis were combined to assess whether NUP62 was a protective or risk factor, resulting in the creation of high-confidence survival landscapes for NUP62. Additionally, the “forestplot” R package was utilized for visual analysis of COX survival data.

### 2.4 Subtyping of NUP62 and immunotherapy analysis

Researchers conducted extensive immunogenomic analysis on over 10,000 tumor samples encompassing 33 different cancer types from the TCGA database. Across tumor types, they successfully identified six immune subtypes by evaluating macrophage or lymphocyte markers, Th1 to Th2 cell ratios, the range of genetic heterogeneity among tumors, aneuploidy, neoantigen burden range, total cell landscape, immune-regulatory gene expression, and prognosis. The six subtypes are introduced as follows:

C1 (Tissue Healing) subtype exhibits elevated angiogenic gene expression, a high proliferation rate, and significant acquired immune infiltration with a Th2 bias. Primary associated cancers include colorectal adenocarcinoma (COAD), rectal adenocarcinoma (READ), lung squamous cell carcinoma (LUSC), luminal A subtype of breast invasive ductal carcinoma (BRCA), classical subtype of head and neck squamous cell carcinoma (HNSC), and chromosomally unstable gastrointestinal subtype.

C2 (IFN-γ Dominant) subtype demonstrates the highest M1/M2 macrophage polarization, strong CD8 signaling, and, like the C6 subtype, the highest TCR diversity. Additionally, the C2 subtype has a high proliferation rate, with primary associated cancers including hypermutated BRCA, gastric cancer, ovarian cancer, HNSC, and cervical squamous cell carcinoma and endocervical adenocarcinoma (CESC).

C3 (Inflammatory) subtype shows elevated Th17 and Th1 gene expression but fails to effectively inhibit tumor cell proliferation. Like the C5 subtype, C3 has fewer aneuploidies and overall cellular copy number alterations compared to other subtypes. Primary associated cancers include most kidney cancers, prostate adenocarcinoma (PRAD), pancreatic adenocarcinoma (PAAD), and thyroid papillary carcinoma (THCA).

C4 (Lymphocyte-Depleted) subtype is characterized by prominent macrophage features, Th1 suppression, and high M2 responsiveness. Primary associated cancers include specific subtypes of adrenocortical carcinoma (ACC), pheochromocytoma and paraganglioma (PCPG), hepatocellular carcinoma (LIHC), and gliomas.

C5 (Immunologically Silent) subtype has the lowest lymphocyte count, the highest macrophage response, and is dominated by M2 macrophages. This subtype is primarily a subtype of low-grade gliomas (LGG) of the brain, including glioma CpG island methylator phenotype-high (CIMP-H), 1p/19q codeleted subtype, and fibrillary astrocytoma-like (PA-like) type. Additionally, the remaining types are mainly within the C4 subtype, while isocitrate dehydrogenase mutant types are more prevalent in the C5 subtype than in the C4 subtype.

C6 (TGF-β Dominant) subtype is a smaller group composed of a mix of cancers and does not dominate any TCGA subtype. This subtype has the highest TGF-β signature and high lymphatic infiltration, with equal distribution of Type I and Type II T cells.

This study further explored the relationship between NUP62 and patient prognosis in external datasets using the BEST database and investigated the correlation between NUP62 expression and the immunotherapy response in cancer patients (30).

### 2.5 Pathway and mechanism of action analysis

To delve into the mechanism of action of NUP62-related pathways, we classified various tumor samples based on NUP62 expression levels (the top 30% as the high-expression group and the bottom 30% as the low-expression group). Subsequently, we employed Gene Set Enrichment Analysis (GSEA) to investigate the differential activation or inhibition states of 50 characteristic genomic signatures and 83 metabolic genomic signatures between the high and low NUP62 expression groups across different malignancies. Utilizing the “GSVA” R package, we quantitatively analyzed 14 functional status genomic signatures using the “z-score” algorithm. Based on the z-score values of each genomic signature, we conducted subsequent data processing and analysis. Furthermore, we applied “Pearson” correlation analysis to assess the statistical correlation between the z-score of each genomic signature and NUP62 expression levels. Additionally, we identified genes that underwent significant changes in both high and low NUP62 expression groups.

### 2.6 Identification of chemicals interacting with NUP62

To explore the potential association between NUP62 expression and drug sensitivity, we leveraged the GSCA database (http://bioinfo.life.edu.cn/web/GSCALite/). Through the GSCA platform, we obtained information on small molecule drugs from the GDSC and CTRP databases. Furthermore, we identified genes differentially expressed between the high and low NUP62 expression groups across various malignancies. To screen for biomarkers closely related to NUP62, we selected the top 150 significantly upregulated and downregulated genes. Simultaneously, we downloaded the CMAP_gene_signatures RData file, which contains 1,288 attributes associated with compound functional characteristics.

## 3 Results

### 3.1 Expression and mutation of NUP62 in humans

**Figure 1:**
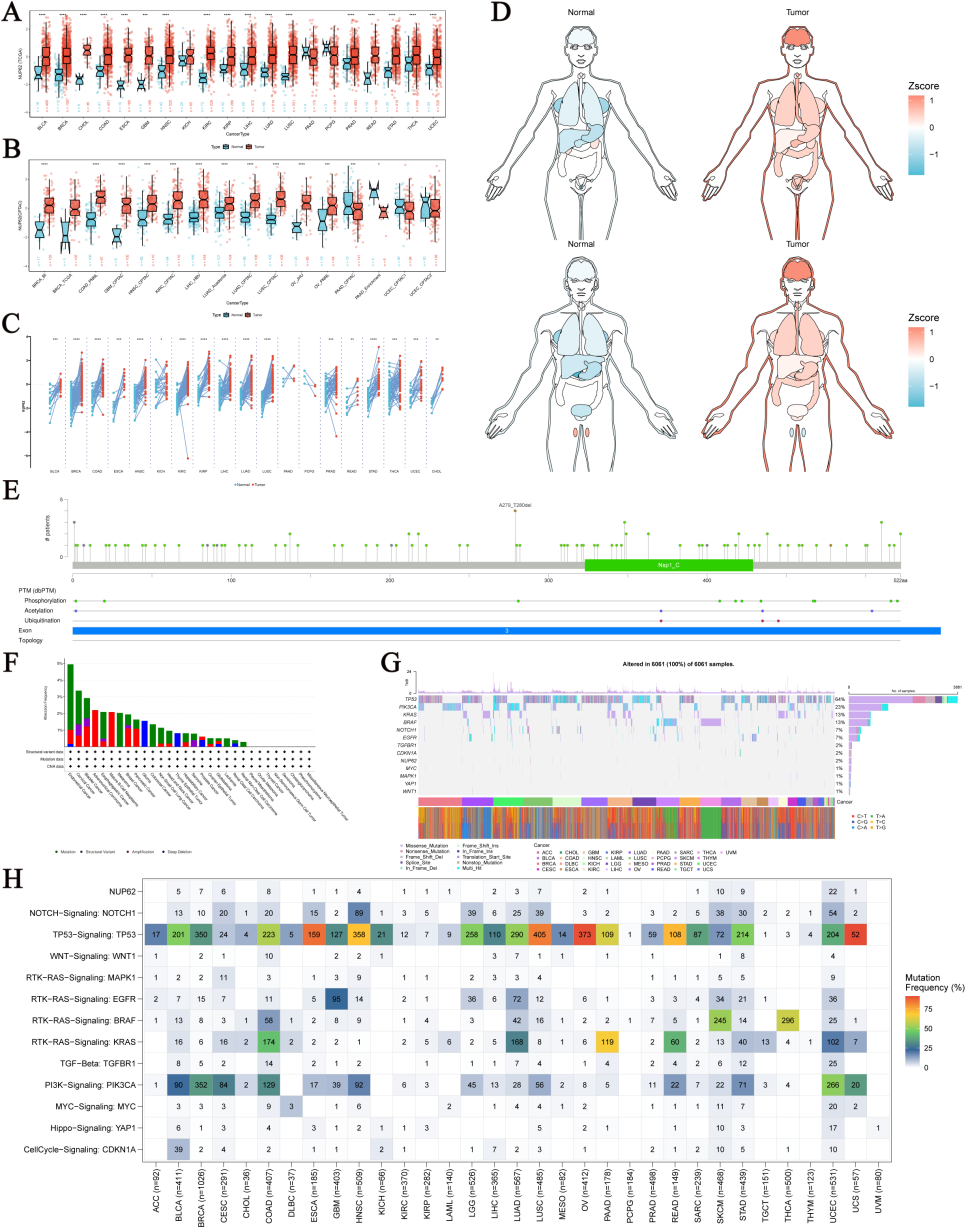
Expression and Mutation of NUP62. **(A)** Expression of NUP62 mRNA in tumor and normal tissues. **(B)** Expression of NUP62 in tumor and normal tissues from the CPTAC database. **(C)** Expression of NUP62 in tumor and paired adjacent normal tissues. **(D)** Expression and distribution of NUP62 in organs of tumor and normal tissues. **(E)** Mutation sites of NUP62. **(F)** Mutation frequencies and corresponding mutations of NUP62 in different cancers. **(G)** Gene alteration sites and numbers of NUP62 in different cancers. **(H)** Distribution of NUP62 and other signals in cancers. *P<0.05. **P<0.01. ***P<0.001. ****P<0.0001.

Using unpaired and paired sample data from the TCGA database, we conducted an in-depth analysis at the mRNA level and observed an upward trend in NUP62 expression across multiple cancer types, including Bladder Urothelial Carcinoma (BLCA), Breast Invasive Carcinoma (BRCA), COAD, Esophageal Carcinoma (ESCA), HNSC, Kidney Renal Clear Cell Carcinoma (KIRC), Kidney Renal Papillary Cell Carcinoma (KIRP), LIHC, Lung Adenocarcinoma (LUAD), LUSC, and Stomach Adenocarcinoma (STAD) (**Figures 1A, C**). Additionally, we further validated these findings in the CPTAC database (**Figure 1B**). **Figure 1D** depicts the expression profile of NUP62 in different human organs. Notably, NUP62 expression levels were significantly upregulated in most cancer types, while they exhibited a downward trend in testicular tissue. NUP62 displayed variable expression patterns in most cancers, with mutation sites distributed as shown in **Figure 1E**. Leveraging the cBioPortal (TCGA, Pan-cancer Atlas) database, we comprehensively assessed the pan-cancer mutational characteristics of the NUP62 gene. The results indicated that the most common types of variations in NUP62 included mutations, amplifications, and deep deletions, with Endometrial Cancer, Cervical Cancer, and Bladder Cancer having the highest mutation rates (**Figure 1F**). Subsequently, we conducted an in-depth analysis of the distribution characteristics of NUP62 mutations across different cancers and explored the types of single nucleotide variants (SNVs). The results showed that missense mutations dominated among various types of variations (**Figure 1G**). Finally, we conducted a comprehensive analysis of the distribution of NUP62 and other genes in cancer samples. The results revealed that among all cancer types, NUP62 was most widely distributed in UCEC, while the TP53 signal was significantly distributed in most cancers (**Figure 1H**).

### 3.2 Expression of NUP62 in cells

Based on fluorescence images provided by the Human Protein Atlas (HPA), we observed that NUP62 is primarily localized within the cell nucleus (**Supplementary Figure 1A**). Researchers systematically analyzed normal tissues such as the cerebral cortex, liver, colon, kidney, and pancreas, as well as tumor tissues such as lung cancer, liver cancer, colorectal cancer, pancreatic cancer, and breast cancer, using immunohistochemical techniques. The results indicated that compared to normal tissues, the expression level of NUP62 in tumor tissues exhibited a significant upward trend (**Supplementary Figure 1B**).

### 3.3 Correlation between NUP62 and clinical characteristics across various cancer types

**Figure 2:**
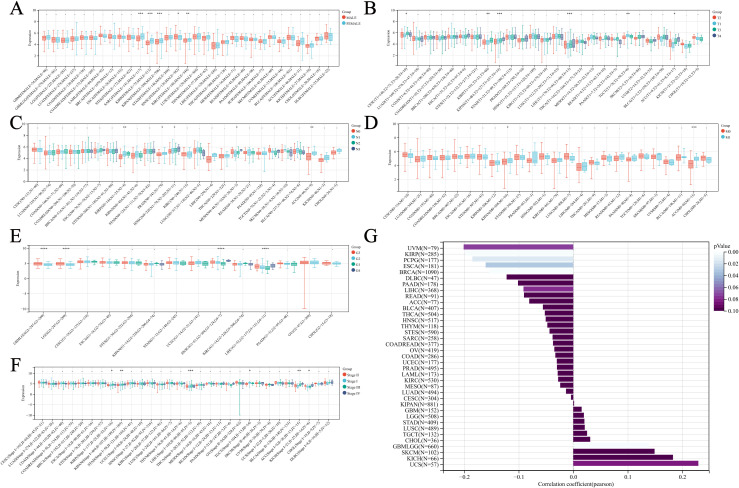
Relationship between NUP62 Expression and Clinical Characteristics Across Various Cancer Types. **(A)** Correlation between gender and NUP62 expression in pan-cancer. **(B)** Correlation between NUP62 expression and T stage in pan-cancer. **(C)** Correlation between NUP62 expression and N stage in pan-cancer. **(D)** Correlation between NUP62 expression and M stage in pan-cancer. **(E)** Correlation between NUP62 expression and G stage in pan-cancer. **(F)** Correlation between NUP62 expression and tumor stage in pan-cancer. **(G)** Correlation between age and NUP62 expression. *P<0.05. **P<0.01. ***P<0.001. ****P<0.0001.

As shown in **Figure 2A**, gender differences in NUP62 expression levels are observed in sarcoma (SARC), KIRP, and the Pan-kidney cohort (KIPAN), with significantly higher expression in female patients compared to male patients. Additionally, the expression level of NUP62 is closely related to age. In KIRP, PCPG, and ESCA, there is a negative correlation between age and NUP62 expression (**Figure 2G**). Furthermore, the expression of NUP62 is associated with various cancer stages. In KIPAN and LIHC, differences in NUP62 expression are observed among tumor patients with different T stages (**Figure 2B**). In KIRP and ACC, NUP62 expression exhibits certain differences across different N stages (**Figure 2C**). Only in ACC is there an association between NUP62 expression and its M stage (**Figure 2D**). In glioma (GBMLGG), LGG, HNSC, and LIHC, significant differences in NUP62 expression are found among tumor patients with different G stages (**Figure 2E**). In LIHC, NUP62 expression is correlated with its stage classification (**Figure 2F**).

### 3.4 Diagnostic value of NUP62 in pan-cancer

**Figure 3:**
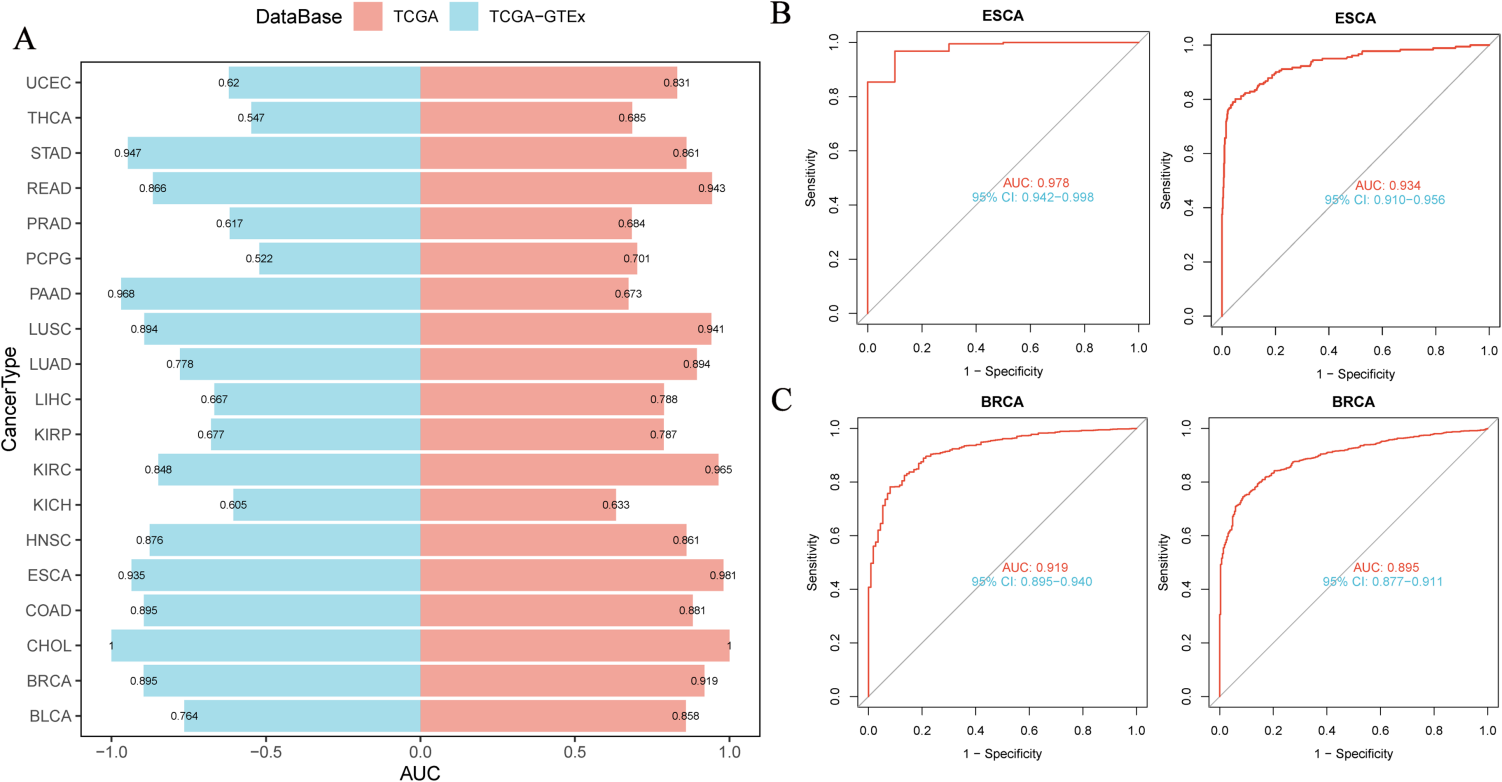
Correlation between NUP62 Expression and Diagnosis of Various Cancer Types. **(A)** Diagnostic value of NUP62 across various cancer types; **(B, C)** ROC curves of NUP62 in ESCA and BRCA.

We evaluated the diagnostic capacity of NUP62 for various cancers in both the TCGA dataset and the combined TCGA-GTEx dataset (**Figure 3A**). The results indicated that the AUC value of NUP62 in ESCA was >0.9, suggesting a high diagnostic value for ESCA. Additionally, the AUC values in BRCA, LUSC, COAD, STAD, READ, KIRC, and HNSC were all between 0.8 and 0.9, indicating that NUP62 also has diagnostic value for these tumors. **Figures 3B, C** depict the ROC curves of NUP62 in ESCA and BRCA, respectively. Therefore, NUP62 is an effective diagnostic biomarker across various cancer types.

### 3.5 Correlation between NUP62 expression and pan-cancer prognosis

**Figure 4:**
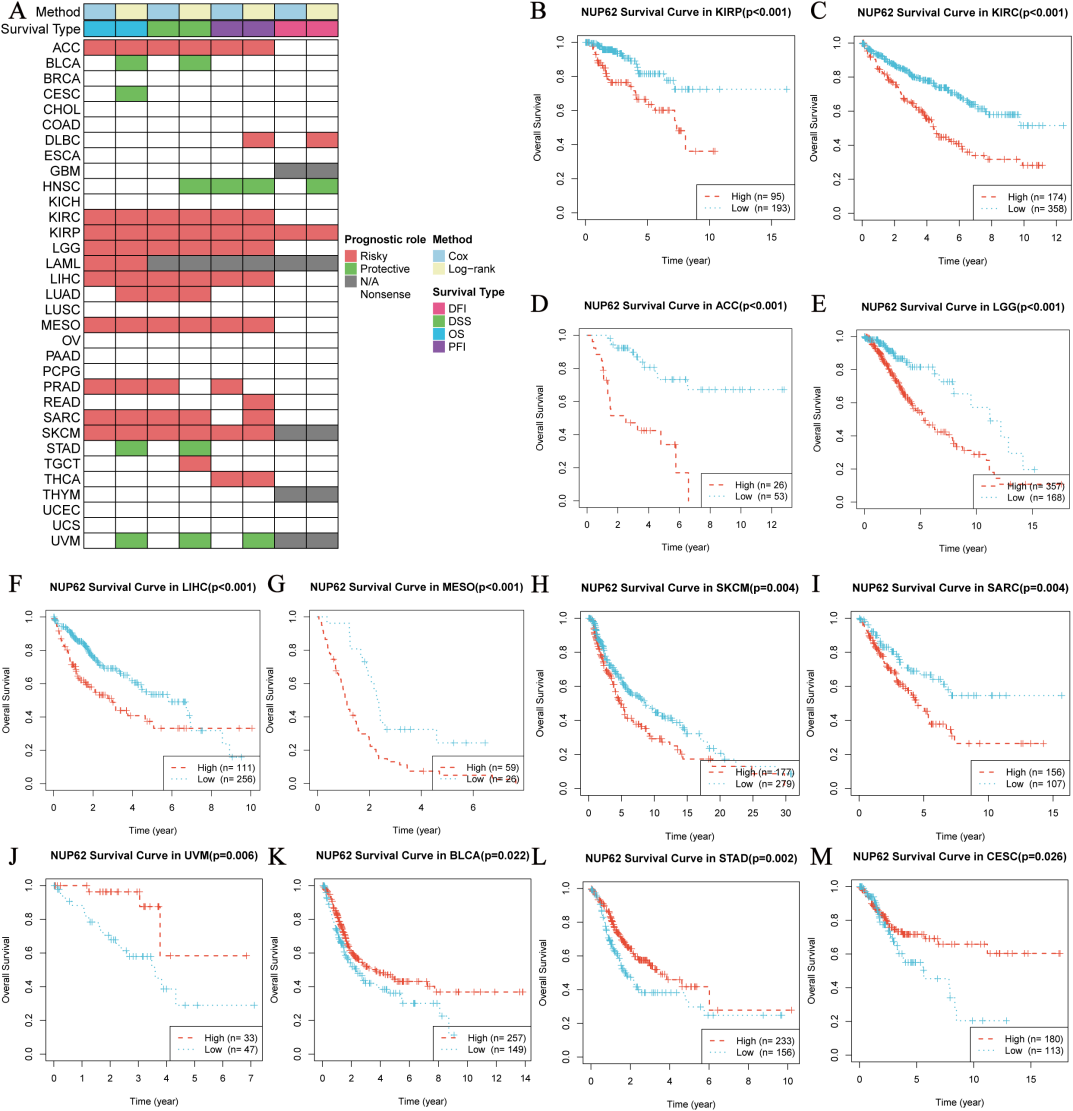
Survival Analysis Spectrum of NUP62 Across Multiple Cancer Types. **(A)** Correlation between NUP62 expression levels and OS, disease-specific survival (DSS), disease-free interval (DFI), and progression-free interval (PFI). **(B-M)** Relationship between NUP62 expression levels and OS in patients with different cancer types.

To gain deeper insights into the clinical significance of NUP62 in the field of cancer, we analyzed its prognostic value in multiple malignant tumors. The results in **Figure 4A** clearly show that NUP62 expression levels are significantly associated with poor prognosis in various cancers, serving as a risk factor for their prognosis. **Figures 4B–M** present the KM survival curves for overall survival (OS) based on NUP62 expression levels in some cancers. The results indicate that high expression of NUP62 is significantly associated with shorter OS in KIRP, KIRC, ACC, LGG, LIHC, mesothelioma (MESO), skin cutaneous melanoma (SKCM), and SARC (p < 0.05). However, in uveal melanoma (UVM), BLCA, STAD, and CESC, high expression of NUP62 is associated with longer survival, potentially playing a protective role.

### 3.6 The role of NUP62 in cancer pathways

**Figure 5:**
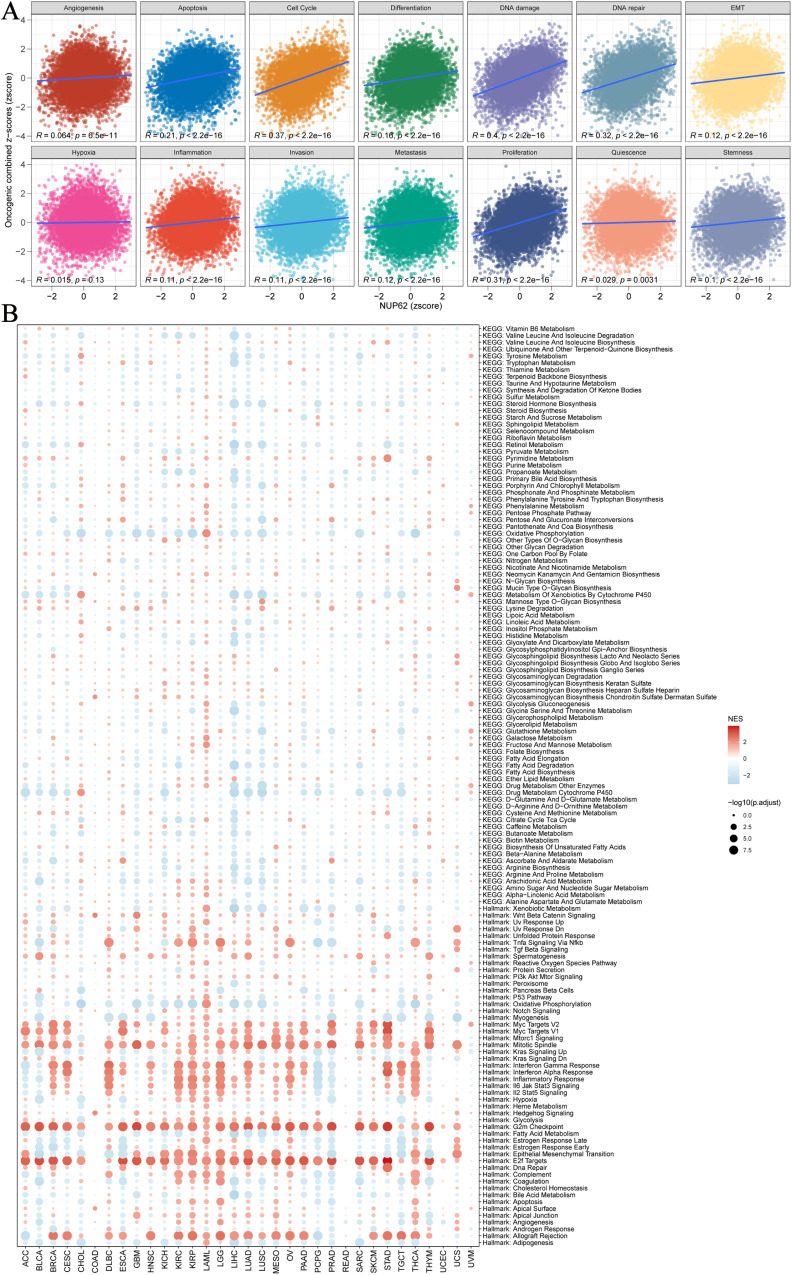
**(A)** Relationships between NUP62 and 14 malignant features of cancer; **(B)** Enrichment differences of NUP62 in 50 HALLMARK and 83 metabolic gene sets.

To delve deeper into the potential roles and specific functions of NUP62 in cancer, we employed an integrated analysis of signature gene expression to assess the activity status of NUP62 pathways. We utilized the z-score parameter from Gene Set Variation Analysis (GSVA) to quantify and evaluate 14 functional status gene sets, covering angiogenesis, apoptosis, cell cycle regulation, cell differentiation, DNA damage response, DNA repair mechanisms, epithelial-mesenchymal transition (EMT), hypoxic adaptation, inflammatory response, invasive behavior, metastatic potential, cell proliferation, cellular quiescence, and cellular stemness features. From this, composite z-scores were obtained. Subsequently, we calculated the Pearson correlation coefficients between NUP62 and each functional status gene set score (**Figure 5A**). The results revealed significant positive correlations between NUP62 expression levels and cell cycle regulation, DNA damage response, and DNA repair mechanisms. Additionally, we collected datasets from cancer patients and conducted Gene Set Enrichment Analysis (GSEA). The analysis results indicated that NUP62 may be involved in various tumor-related pathways and metabolic regulatory processes, such as the G2/M checkpoint, E2F transcription factor target genes, allograft rejection, and mitotic spindle formation (**Figure 5B**).

### 3.7 The relationship between NUP62 and cellular pathways

**Figure 6:**
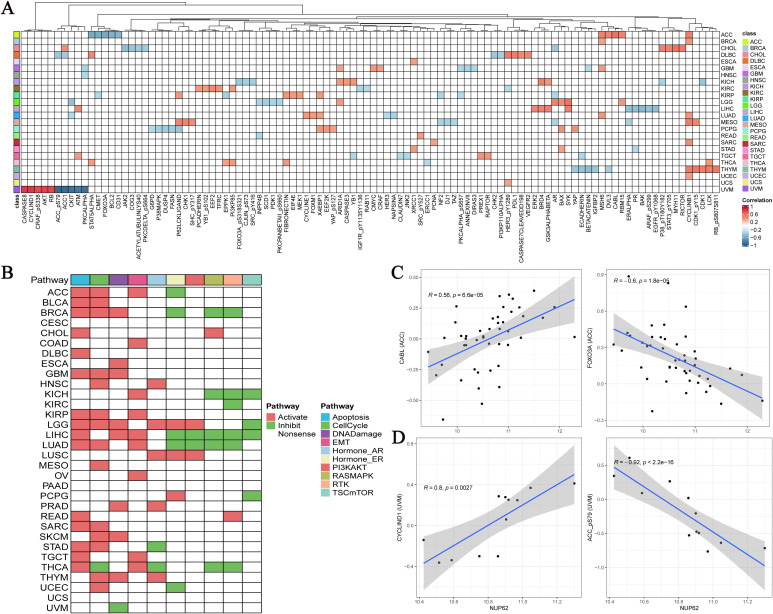
**(A)** Top five functionally related proteins of NUP62 across various cancers in the TCGA database; **(B)** Relationships between cancer and 10 cancer-related pathways; **(C, D)** NUP62-related functional proteins in ACC and UVM from the TCPA database.

Utilizing the TCGA database, we conducted an in-depth exploration of the interaction between NUP62 and functional proteins within the TCGA database, and found that NUP62 exhibits significant correlations with multiple key functional proteins across various cancer types. Specifically, in UVM, the expression level of NUP62 is significantly positively correlated with CASPASE8, CYCLIN D1, CRAF (pS338), AKT, and RB proteins, while it is significantly negatively correlated with the expression levels of ACC-pS79, ACC1, CKIT, ATM, and PKCα. In ACC, the expression of NUP62 is positively correlated with the expression of CABL protein and negatively correlated with the expression of FOXO3A protein (**Figure 6A**). **Figure 6B** illustrates the relationships between cancer and 10 cancer-related pathways, where the activation of apoptosis, activation, cell cycle, and DNA damage promotes the occurrence of cancer. Subsequently, we detailed the most significantly correlated functional proteins of NUP62 in ACC and UVM from the TCPA database (**Figures 6C, D**).

### 3.8 The relationship between NUP62 and immune subtypes and immunotherapy

**Figure 7:**
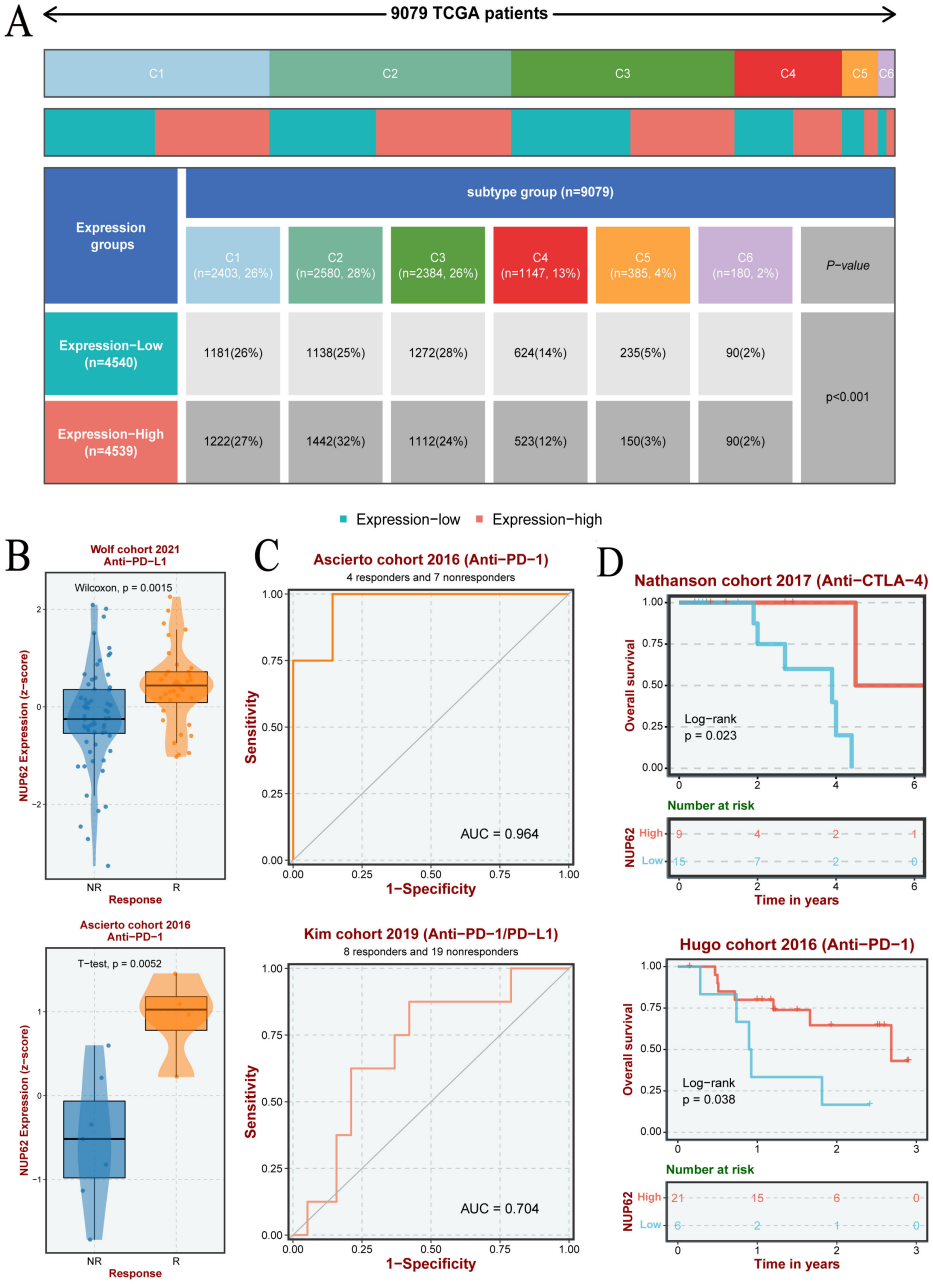
**(A)** Differences in the number of pan-cancer immune subtypes between the high and low NUP62 expression groups. **(B)** Expression of NUP62 in responders and non-responders within different immunotherapy cohorts. **(C)** Diagnostic value of NUP62 in different immunotherapy settings. **(D)** Correlation between NUP62 expression and survival rates in different immunotherapy cohorts.

Based on nearly 10,000 cancer samples from the TCGA database, we subclassified them into six distinct immune subtypes. The analysis revealed that C1, C2, C3, and C4 subtypes dominate among all immune subtypes. Additionally, in cancer samples with high NUP62 expression, the proportion of the C2 subtype was significantly higher than that in samples with low NUP62 expression; conversely, in patients with low NUP62 expression, the proportions of C3 and C5 subtypes were significantly higher than those in patients with high NUP62 expression (**Figure 7A**). Subsequently, we delved into the correlation between NUP62 expression levels and immunotherapy response in clinical trials. Among the two immunotherapy cohorts included in the study, patients with high NUP62 expression had a significantly higher response rate to immunotherapy than those with low expression (**Figure 7B**). Notably, we evaluated the potential efficacy of NUP62 as a predictor of immunotherapy response using ROC curve analysis (**Figure 7C**). By comparing the impact of two different immunotherapy regimens on patients’ overall survival, we found that patients with elevated NUP62 expression had a significantly longer overall survival compared to those with decreased NUP62 expression (**Figure 7D**).

### 3.9 The relationship between NUP62 and immunity

**Figure 8:**
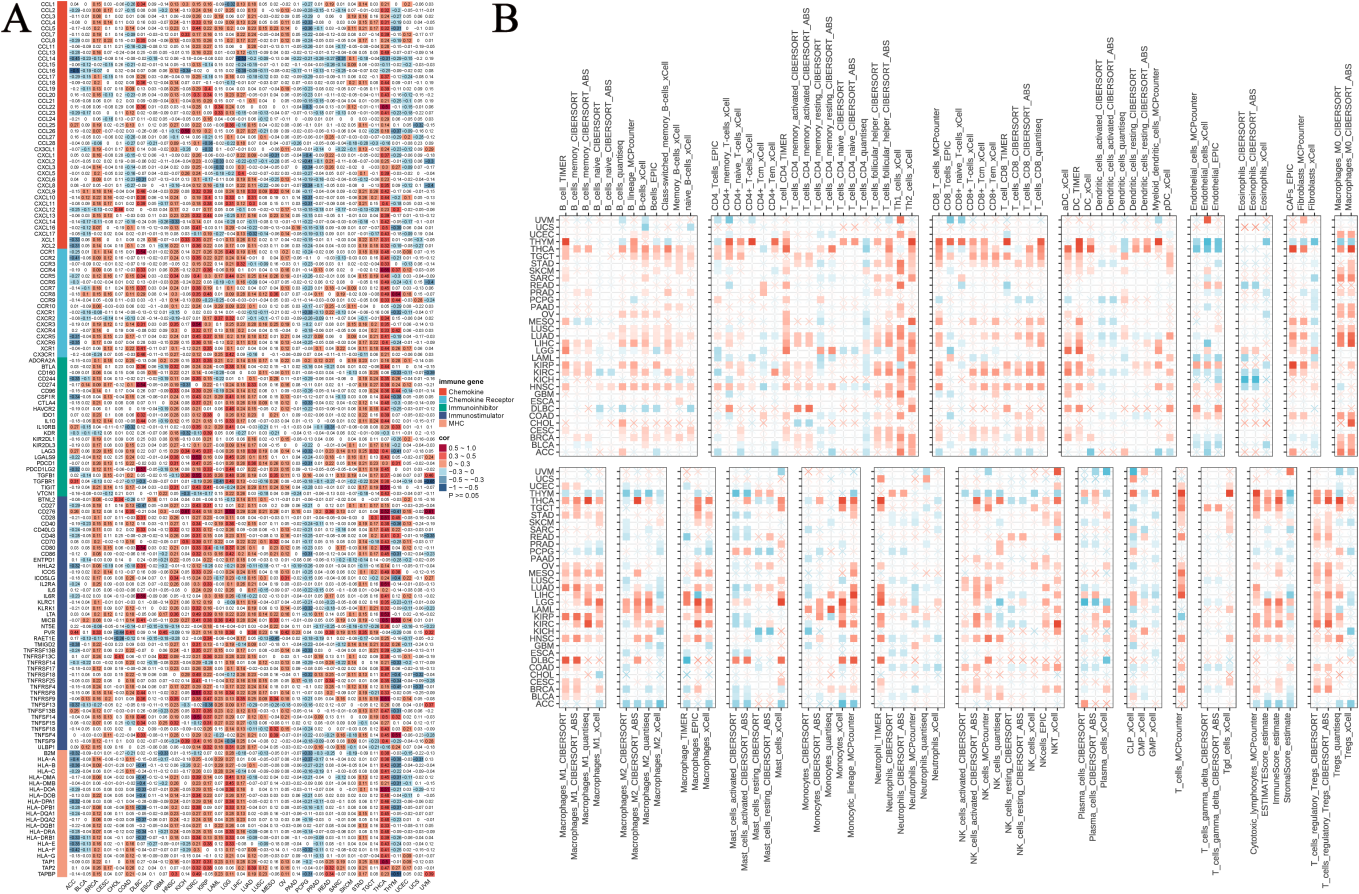
The relationship between NUP62 expression and immune infiltration. **(A)** Heatmap showing the correlation between NUP62 mRNA expression and the expression of chemokines, chemokine receptors, immune inhibitors, immune stimulators, and MHC genes. **(B)** Correlation between NUP62 expression and tumor immune infiltration.

Immunotherapy has emerged as a primary modality in cancer treatment, prompting our investigation into the potential association between NUP62 and cancer immunity. Our results indicate that across various cancer types, the expression level of NUP62 significantly correlates with the expression of immune-related genes, such as those in THCA, KIRC, KIRP, and PCPG (**Figure 8A**). Furthermore, by examining the levels of immune cell infiltration in the TME, we found that the expression of NUP62 is significantly correlated with the infiltration levels of multiple immune cell types, including Th1 cells, Th2 cells, dendritic cells (DC-TIMER), M0 and M1 macrophages (Macrophages-M0-CIBERSORT), neutrophils (neutrophil-TIMER), T cells (T-cells-MCPcounter), and regulatory T cells (T-cells-regulatory-CIBERSORT), among others, across several cancer types (**Figure 8B**). These findings suggest that NUP62 may play a crucial role in the tumor immune microenvironment, potentially affecting tumor growth and the efficacy of immunotherapy by regulating the expression of immune cells or immune-modulating genes.

### 3.10 NUP62 may influence chemotherapy response

**Figure 9:**
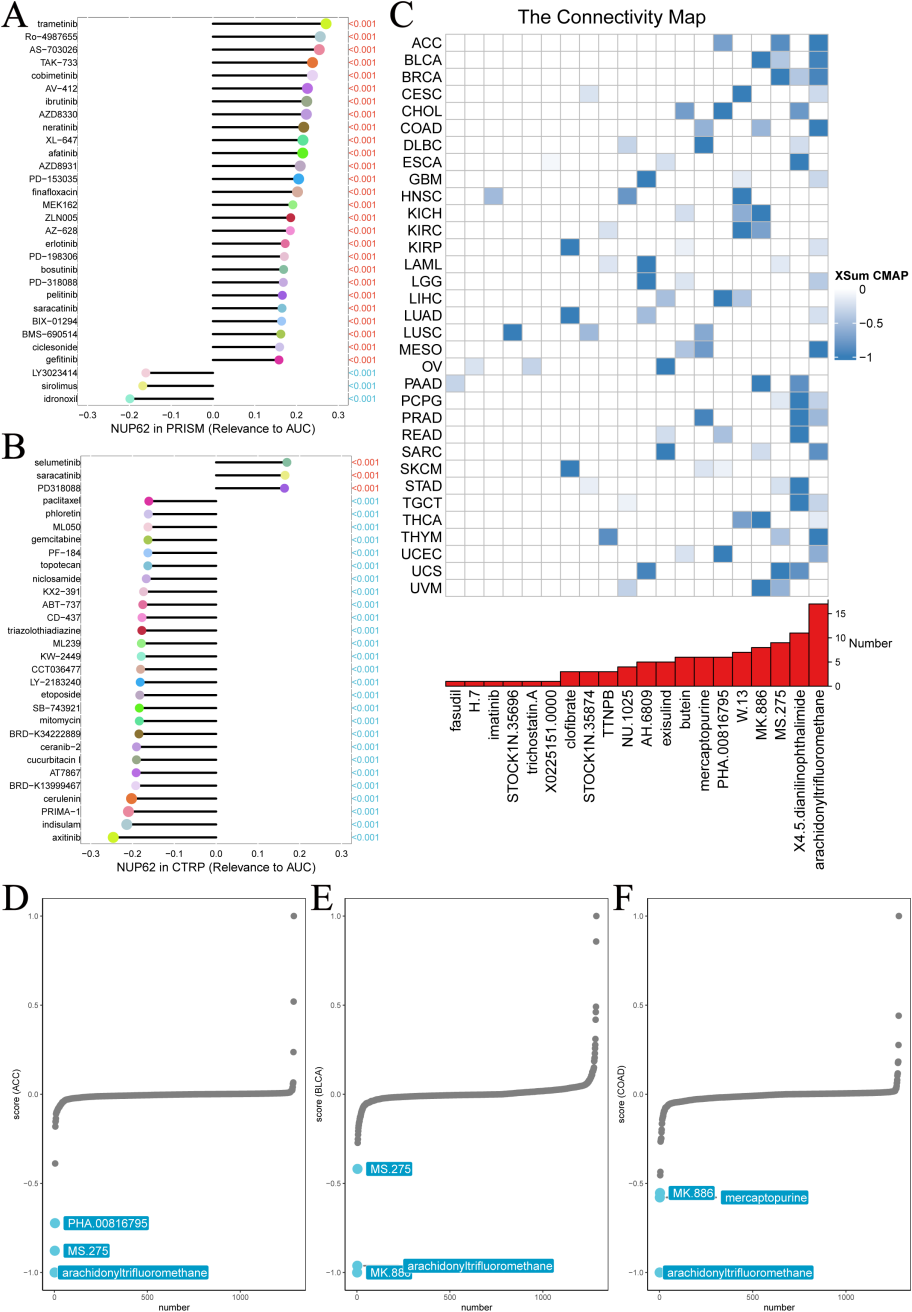
Chemotherapy resistance analysis. Two different databases were utilized to investigate the association between NUP62 expression and drug sensitivity. **(A)** PRISM. **(B)** CTRP. **(C)** Identification of NUP62-targeting compounds through cMap analysis. **(D-F)** NUP62-targeting compounds with clinical significance.

We further delved into the potential association between NUP62 expression levels and drug sensitivity. Results from both PRISM and CTRP drug sensitivity experiments revealed a significant correlation between NUP62 mRNA expression levels and drug sensitivity. The PRISM drug sensitivity experiment showed that the top three drugs positively correlated with NUP62 expression levels were trametinib, Ro-4987655, and AS-703026, while the top three negatively correlated drugs were idronoxil, sirolimus, and LY3023414 (**Figure 9A**). Similarly, the CTRP drug sensitivity experiment also demonstrated that the top three drugs positively correlated with NUP62 expression levels were selumetinib, saracatinib, and PD318088, while the top three negatively correlated drugs were axitinib, indisulam, and PRIMA-1 (**Figure 9B**). The discovery of compounds that can significantly modulate NUP62 activity holds important potential value for developing novel and effective tumor treatment regimens. We found that arachidonyltrifluoromethane significantly impacts NUP62 expression across multiple tumor types (**Figure 9C**). Subsequently, we detailed the specific effects of various compounds on NUP62 in different tumor types and highlighted the significant diagnostic and prognostic predictive value of NUP62 in these tumor types (**Figures 9D–F**).

### 3.11 Validation of NUP62 expression levels in gastric cancer

**Figure 10:**
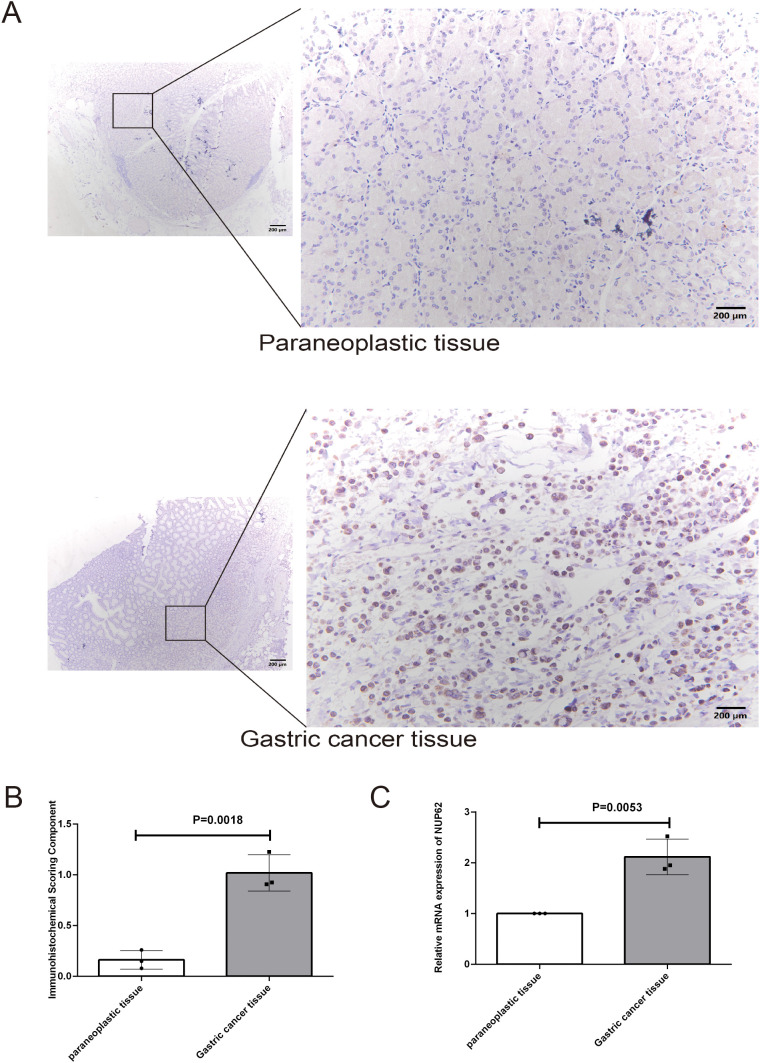
**(A, B)** Represent the protein expression levels of NUP62 in gastric cancer tissues and their adjacent non-cancerous tissues. **(C)** Illustrates the mRNA expression levels of NUP62 in gastric cancer tissues versus their adjacent non-cancerous tissues.

To validate our initial hypothesis, we employed immunohistochemical techniques to conduct an in-depth analysis of NUP62 protein expression levels in gastric cancer tissues and their adjacent non-cancerous tissues. **Figure 10A** visually demonstrates the distribution and intensity of NUP62 expression in both non-cancerous and gastric cancer tissues. The research findings indicate that the expression level of NUP62 in gastric cancer tissues is significantly higher than that in their adjacent non-cancerous tissues (see **Figure 10B**). Furthermore, through quantitative PCR analysis, we found that the mRNA expression level of NUP62 in gastric cancer tissues is also significantly elevated compared to their adjacent non-cancerous tissues.

## 4 Discussion

Firstly, our study determined the expression and mutation status of NUP62 in humans. The results indicated that NUP62 expression is generally higher in cancer cells compared to normal cells. Mutations in NUP62 within tumors may lead to functional abnormalities of NUP62, affecting normal cellular functions and playing a role in tumorigenesis and progression. HPA fluorescence images and staining data demonstrated that NUP62 is primarily localized in the nucleus, consistent with its role as a component of the NPC (20, 31). Further stratification according to patients’ clinical characteristics revealed that NUP62 is associated with the stages of multiple cancers, suggesting its role in tumor progression, invasion, and metastasis (32–34). The expression of NUP62 is also closely related to gender and age, which may be associated with hormones, cellular aging, decreased DNA damage repair capacity, and increased cancer risk (35–37). In the TCGA dataset and the combined TCGA-GTEx dataset, NUP62 exhibited high AUC values across various cancers. Notably, in ESCA, the AUC value was >0.9, indicating that NUP62 has significant value for the screening and diagnosis of multiple cancers, particularly ESCA (38–40). Next, our research found that NUP62 expression is associated with the prognosis of cancer patients, with differential expression observed in different types of cancer patients, as confirmed by KM curves. This suggests that NUP62 can serve as a prognostic marker for some cancers to guide the selection of treatment regimens for cancer patients, taking into account individual differences and formulating personalized treatment plans (41–43). Further GSEA analysis revealed that NUP62 is significantly associated with the cell cycle, DNA damage, and DNA repair, which may be related to NUP62’s involvement in numerous tumor-related pathways and metabolic processes, including the G2/M checkpoint, E2F target genes, allograft rejection, and mitotic spindle. These pathways and processes play crucial roles in cancer cell proliferation, invasion, metastasis, and drug resistance, emphasizing the complexity and diversity of NUP62 in tumors (44–48). Subsequently, we investigated the relationship between NUP62 and TCPA functional proteins, further demonstrating the important role of NUP62 in regulating the cell cycle of cancer patients.

We noted a decreasing trend in the expression level of NUP62 in testicular cancer tissues, which contrasts with the upregulation of NUP62 expression in most cancers. This difference in expression patterns may reflect the unique biology of testicular cancer. Therefore, we believe that testicular cancer is a validated experimental subject worthy of further study. By deeply exploring the expression regulation mechanism of NUP62 in testicular cancer, it is expected to provide new ideas and methods for the diagnosis and treatment of testicular cancer. We have not validated this for the time being, but it will be an important direction for future research.

Although our study found that high expression of NUP62 was associated with longer survival in these tumors, this does not mean that NUP62 is necessarily protective in these tumors. It is possible that the expression level of NUP62 is affected by a variety of factors, which may have different roles in different types of tumors. Therefore, the specific mechanism of action of NUP62 in these tumors and its interrelationship with other biomarkers can be further explored in subsequent studies, with the aim of providing more accurate targets for the treatment of these tumors.

Nuclear pore complex core proteins were shown to be extensively involved in metabolic reprogramming, such as NUP37, Nup210 (49–51). Therefore we speculated that NUP62 might be involved in metabolic pathways in tumors. In the subsequent analysis, we comprehensively analyzed the metabolic pathways in which NUP62 might be involved in pan-cancer by performing GSEA analysis on the KEGG metabolic gene set. The results indicated that NUP62 may inhibit multiple metabolic pathways in most tumors, such as steroid hormone synthesis, fatty acid synthesis, and glucose metabolism. This finding reveals a novel function of NUP62 as a nuclear pore complex core protein in tumor metabolism. Traditionally, NUP62 is mainly thought to be involved in the regulation of nuclear-plasmic transport and cellular signaling, whereas this study links it to metabolic pathways in tumors, providing a new perspective for understanding the role of NUP62 in tumorigenesis and progression.

Simultaneously, our research also revealed that the expression of NUP62 is significantly correlated with the infiltration levels of various immune cells, indicating that NUP62 can regulate the tumor immune microenvironment by influencing immune cells (52–55).

We delved into the correlation between NUP62 expression levels and immunotherapy response. The results showed that in clinical trials of immunotherapy, the response rate to immunotherapy was significantly higher in patients with high NUP62 expression than in patients with low expression. This suggests that NUP62 may be an important biomarker for predicting response to immunotherapy (56). By detecting patients’ NUP62 expression levels, physicians may be able to more accurately predict which patients are more likely to benefit from immunotherapy, thereby optimizing the treatment regimen and improving the therapeutic efficacy. The results of the ROC curve analysis showed the potential efficacy of NUP62 in predicting immunotherapy response, which provides a new way of thinking for the development of immunotherapy evaluation methods (57). Traditional immunotherapy assessment methods mainly rely on indicators such as changes in tumor size, but these indicators often suffer from lag and inaccuracy. In contrast, biomarker-based prediction methods may be able to assess immunotherapy effects earlier and more accurately, thus guiding the adjustment and optimization of treatment regimens (58, 59). The study also found that the overall survival of patients with high NUP62 expression in immunotherapy was significantly longer than that of patients with low expression. This suggests that NUP62 may be associated with immunotherapy resistance (60). Through in-depth study of the mechanism of NUP62’s role in immunotherapy resistance, it may provide new strategies and methods for overcoming immunotherapy resistance, thereby improving the effectiveness of immunotherapy and patient survival (61, 62).

The significant negative correlation of NUP62 with CTLA-4 may imply that the low expression of NUP62 in these two cancers is associated with an attenuated immunosuppressive state (63). This suggests that NUP62 expression in these cancers may not be the primary factor promoting immune evasion or that it interacts in a complex manner with other immune regulatory mechanisms. The significant correlation of NUP62 with the expression of immune-related genes (e.g., THCA, KIRC, KIRP, and PCPG) in multiple cancer types suggests that it may play a role in a wide range of immune regulatory processes (64). This further supports the idea that NUP62 serves as an important node in the cancer immunoregulatory network. Given the significant correlation of NUP62 with immune-related genes, it could be a potential target for immunotherapy (65). By regulating the expression or function of NUP62, it may be able to influence the activity of immune cells and the immune status in the tumor microenvironment, thus providing new strategies for immunotherapy. In the future, further in-depth studies can be conducted to investigate how NUP62 interacts with immune-related genes and how these interactions affect the tumor immune microenvironment and immunotherapeutic effects (66).

The findings suggest that NUP62 expression level may become a marker for predicting the efficacy of specific drugs (67). This means that in future clinical practice, physicians can select the most likely effective drugs based on a patient’s NUP62 expression level, enabling more precise and personalized treatment (68). By understanding the relationship between NUP62 and drug sensitivity, drug developers can design new drugs in a more targeted manner, especially for those drugs that are positively or negatively correlated with NUP62 expression levels (69). This biomarker-based drug development strategy is expected to improve the success rate and clinical application of new drugs. In addition, by monitoring changes in NUP62 expression levels, physicians can make timely adjustments to treatment regimens to avoid or delay the onset of drug resistance. In addition, the efficacy of certain drugs, such as those negatively correlated with NUP62 expression levels, may be affected by NUP62 expression levels, and therefore, other alternative drugs or combination strategies may need to be explored in specific patient populations (70, 71). The present study reveals a novel role of NUP62 in drug sensitivity, which provides important clues for an in-depth understanding of its biological functions and regulatory mechanisms. This can help to further expand the application areas of biomarker research and improve the diagnosis and treatment of diseases such as cancer (72–74).

Additionally, we validated the differential expression of NUP62 between gastric cancer tissues and their adjacent non-cancerous tissues.

In summary, this study revealed the expression characteristics of NUP62 in multiple malignancies and its associations with tumor-related pathways, clinical features, prognosis, and tumor immunity. These findings provide crucial insights into our understanding of the role of NUP62 in tumors and offer new targets for future tumor diagnosis and immunotherapy. However, to fully uncover the mechanisms of NUP62 in tumors, further in-depth research and exploration are required.

## Data Availability

The original contributions presented in the study are included in the article/**Supplementary Files**, further inquiries can be directed to the corresponding authors.

## Supplementary material

Supplementary Figure 1 Expression Levels of NUP62 in Cell Line Models, Cancerous, and Normal Tissues. **(A)** Immunofluorescence images and merged images of the nuclei, NUP62 protein, microtubules, and endoplasmic reticulum (ER) in A-431, U2OS, and U-251MG cell lines obtained from the HPA database. **(B)** IHC images of NUP62 protein expression in normal and tumor tissues obtained from the HPA database.
