# Supplementary figures and images for: Exploring NUP62’s role in cancer progression, tumor immunity, and treatment response: insights from multi-omics analysis
Source: Front Immunol. 2025 Mar 3;16:1559396. doi: 10.3389/fimmu.2025.1559396 (PMC11911477; doi:10.3389/fimmu.2025.1559396)

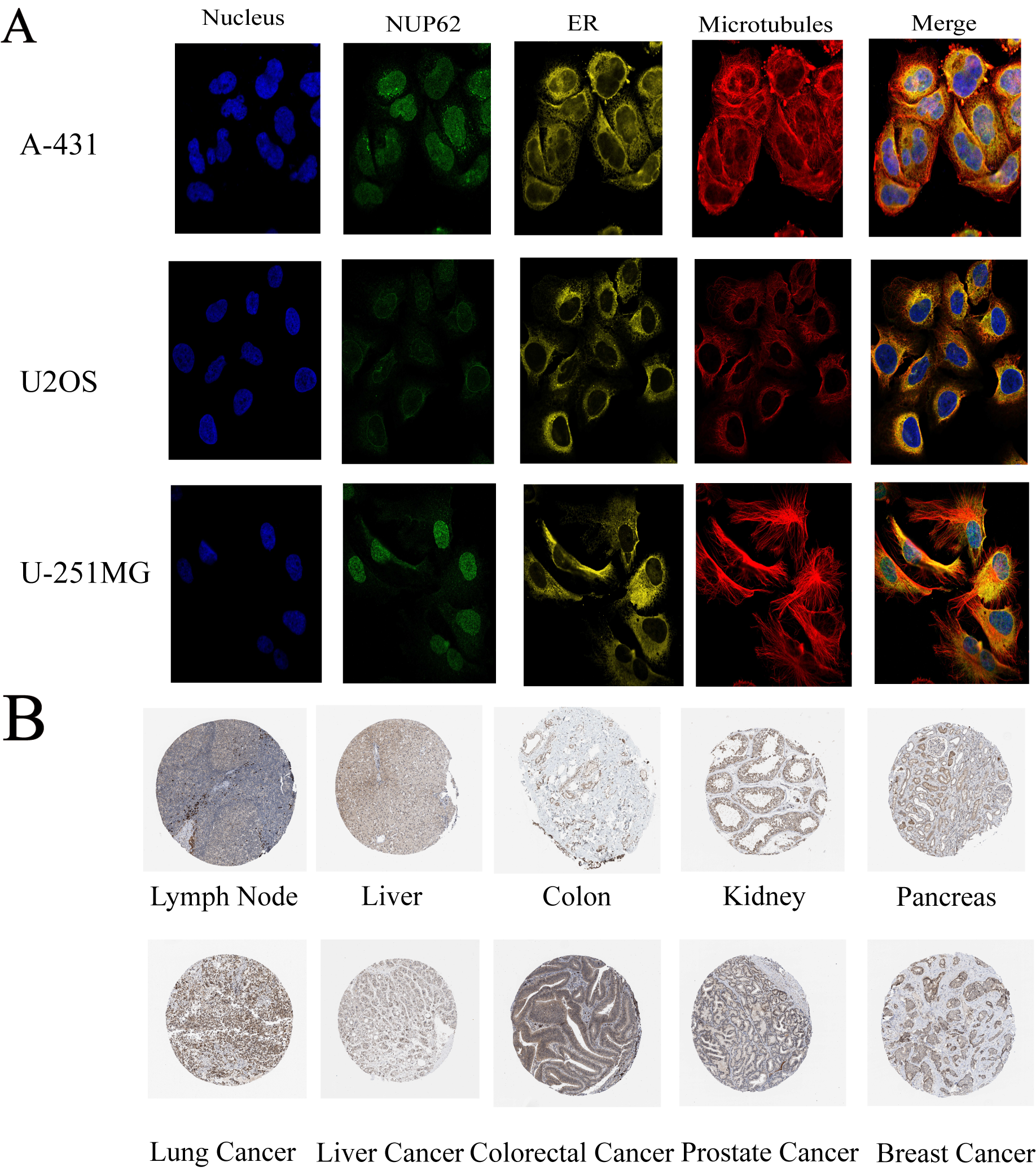

Supplement: Supplementary Figure 1 — Expression Levels of NUP62 in Cell Line Models, Cancerous, and Normal Tissues. (A) Immunofluorescence images and merged images of the nuclei, NUP62 protein, microtubules, and endoplasmic reticulum (ER) in A-431, U2OS, and U-251MG cell lines obtained from the HPA database. (B) IHC images of NUP62 protein expression in normal and tumor tissues obtained from the HPA database. [file Image1.tif]
